# Evidence of D-shaped wounds in the intrasomatic bullet path: two case reports

**DOI:** 10.1007/s12024-022-00538-6

**Published:** 2022-10-14

**Authors:** Luca Tomassini, Anna Maria Manta, Daniele Paolini, Pia Eugenia Ylenia Petrasso, Gianluca Niccolò Piras, Costantino Ciallella

**Affiliations:** Department of Anatomical, Histological, Forensic Medicine and Orthopedic Sciences, Sapienza University of Rome, Rome, Italy

**Keywords:** Atypical gunshot wounds, D-shaped gunshot wounds, Intermediated target, Ricochet, Bullet path

## Abstract

The final appearance of gunshot wounds is influenced by both the projectile’s behavior from the muzzle to the terminal target and by the intrinsic characteristics of the anatomical compartments where the lesion(s) occur. The D-shaped morphology is an uncommon yet well-known finding in the forensic literature and has been described when the surface of impact with the skin is represented by the bullet’s lateral projection. Two cases where D-shaped gunshot wounds were observed are hereby presented: in both cases, interaction with multiple intermediate targets (case 1) and a human intermediate target (case 2) had been documented and confirmed by the forensic examination. Despite the different dynamics of production, this peculiar morphology was described throughout most of the intrasomatic bullet path in both the victims. The discovery of D-shaped gunshot wounds can guide the forensic pathologist in the ballistic reconstruction of the event by supporting the hypothesis of an interaction with an intermediate target that has led to deviation from the initial trajectory and destabilization of the bullet associated with loss of kinetic energy.

## Introduction

Forensic ballistics can be defined as the study of the projectile’s behavior to reconstruct the defining events in the production of a gunshot wound (GSW) and is divided into internal, external, and terminal ballistics [1, 2]. While internal ballistics mostly focuses on the mechanisms of bullet ejection from the inside of the firearm, external ballistics describes the flight from the muzzle to the final target, and terminal ballistics, also referred to as wound ballistics, analyzes the injuries that occur in the different anatomical compartments.

The course of the bullet outside the barrel greatly influences the appearance of the GSW, especially when interaction with interposed objects, i.e., intermediate targets, occurs, which can cause alteration of the angle of incidence, tumbling and yawing of the bullet, resulting in an unstable flight and, ultimately, in atypical entrance wounds [2, 3].

Intermediate targets are environmental objects opposing resistance to the projectile, causing deviation from the original trajectory, decrease in velocity, and so, dispersion of kinetic energy, which can lead to:

- Ricochet, when the angle of incidence is small;
- Bullet deformation and fragmentation;
- Penetration/perforation of the intermediate target [2, 4–7].

The GSWs produced by the modifications in the bullet’s behavior greatly differ from those occurring when the initial trajectory and stability remain unaltered [3, 6, 8, 9]. An infrequently observed morphology of the entrance wound has been described in the literature as D-shaped when the surface of impact with the cutaneous tissue is represented by the lateral projection of the bullet [2, 7, 8, 10, 11].

This peculiar appearance is given by an object with an acute angle at the apex and a recognizable cylindric base with regular margins. In fact, the forensic pathologist examining the wounds needs to interpret this finding and reconstruct the dynamics and the manner of death based on the collection of anatomical and circumstantial evidence. For the sake of the analysis conducted in this small but paradigmatic case series, the Authors relied on the similarities observed during the study of the intrasomatic bullet paths.

Two extremely different dynamics of production of D-shaped GSWs, where interaction with multiple (Case 1) and human (Case 2) intermediate targets was documented, are hereby presented, and analyzed. These examples are exceptional as in both cases the D-shaped conformation was demonstrated throughout the intrasomatic bullet path, from the entrance wound to the affected tissues.

## Case report

### Case 1

#### The incident details and crime scene investigation

**Fig. 1:**
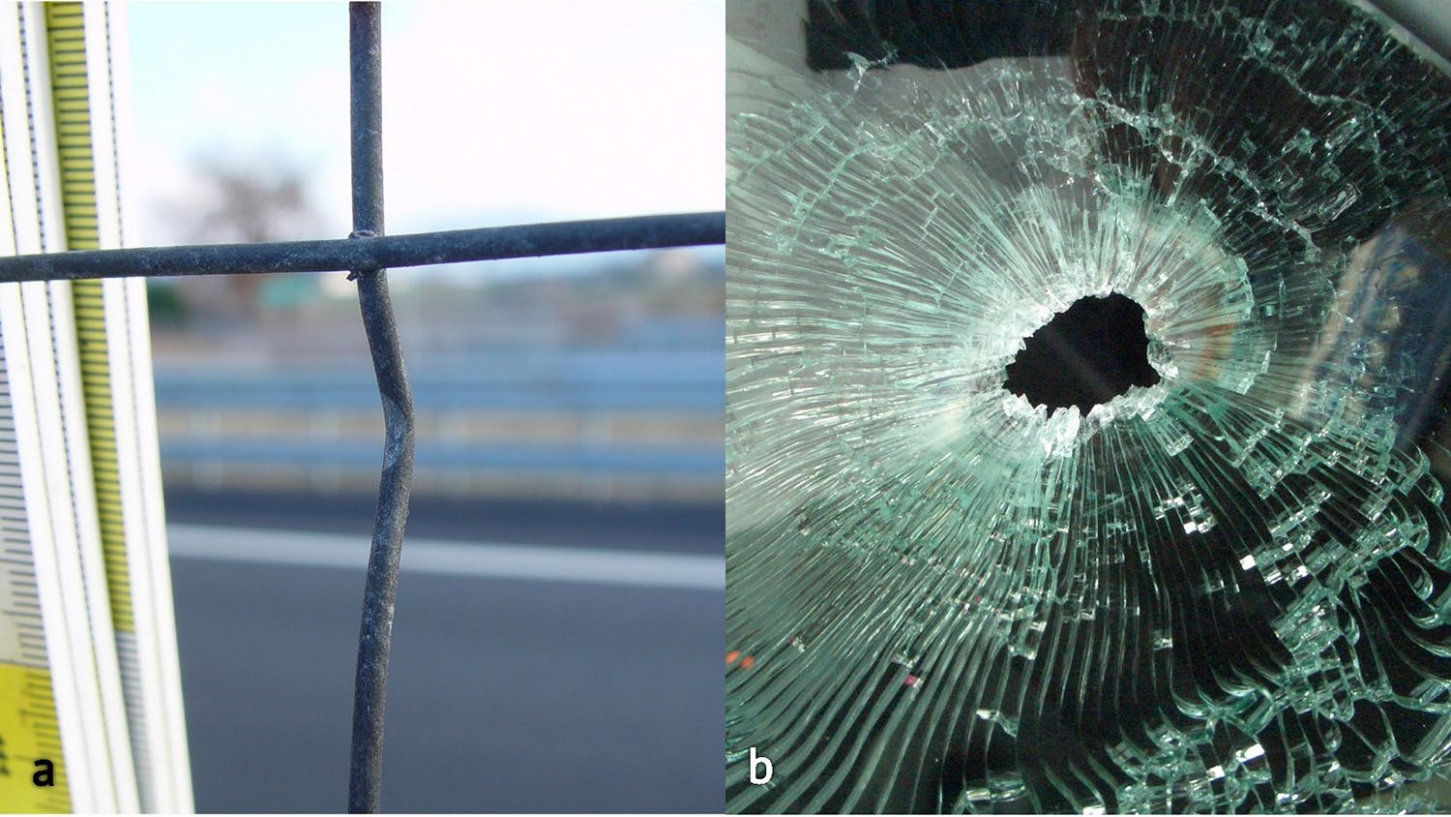
Case 1 intermediate targets. **a** First intermediate target: the wire fence dividing the two carriageways. Interaction with the bullet caused a “watermelon slice”–like deformation of the metal and subsequent ricochet from the original trajectory. **b** Second intermediate target: glass of the rear window. Note the shape of the hole which accurately represents the lateral projection of the bullet

**Fig. 2:**
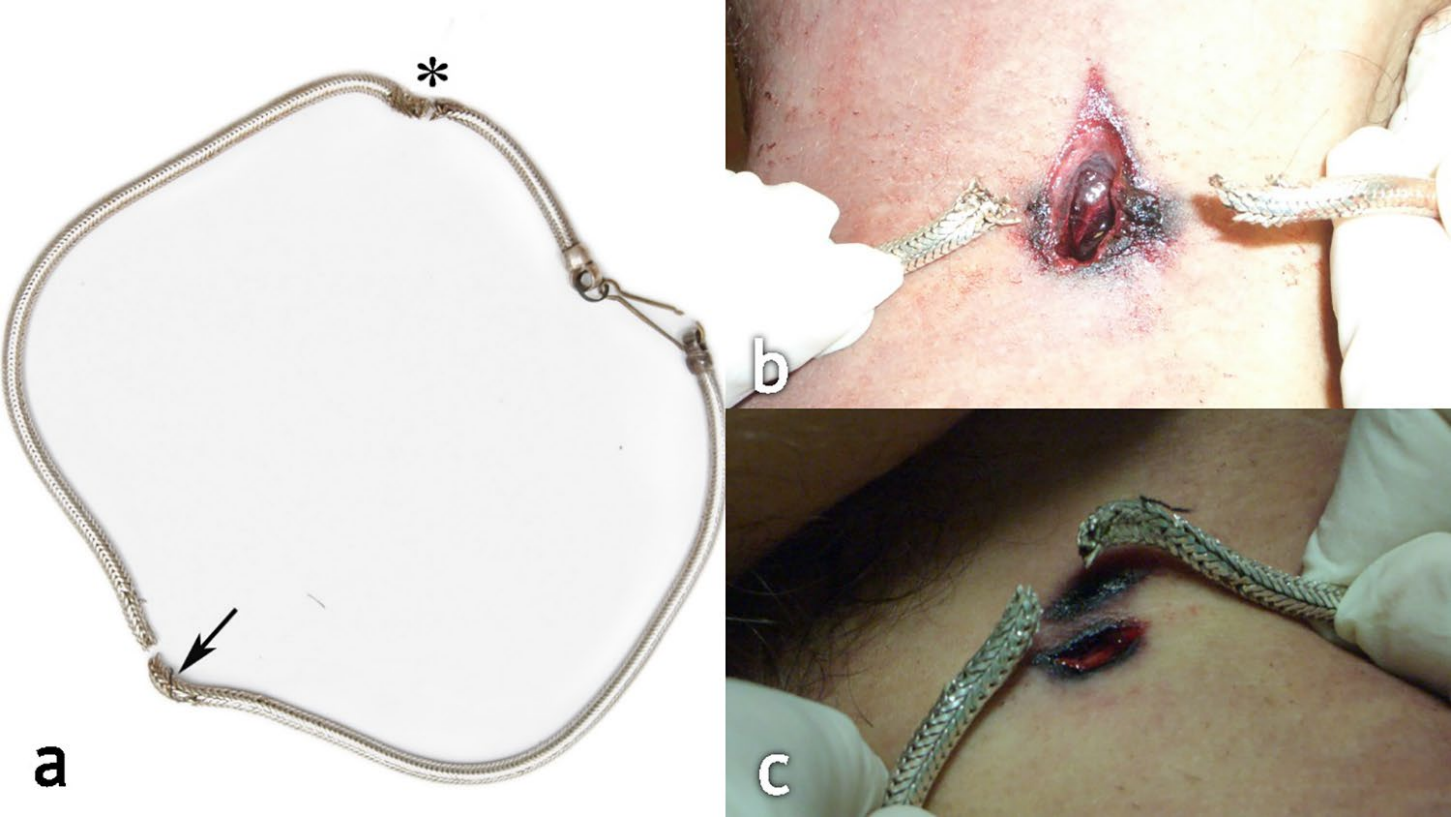
Case 1 third intermediate target. **a** The necklace worn by the victim with two breaks at the level of the entrance (*) and exit (arrow) wounds. **b** Repositioning of the necklace during the autopsy with the margins at the level of the entrance wound. **c** Repositioning of the necklace during the autopsy with the margins at the level of the exit wound

**Fig. 3:**
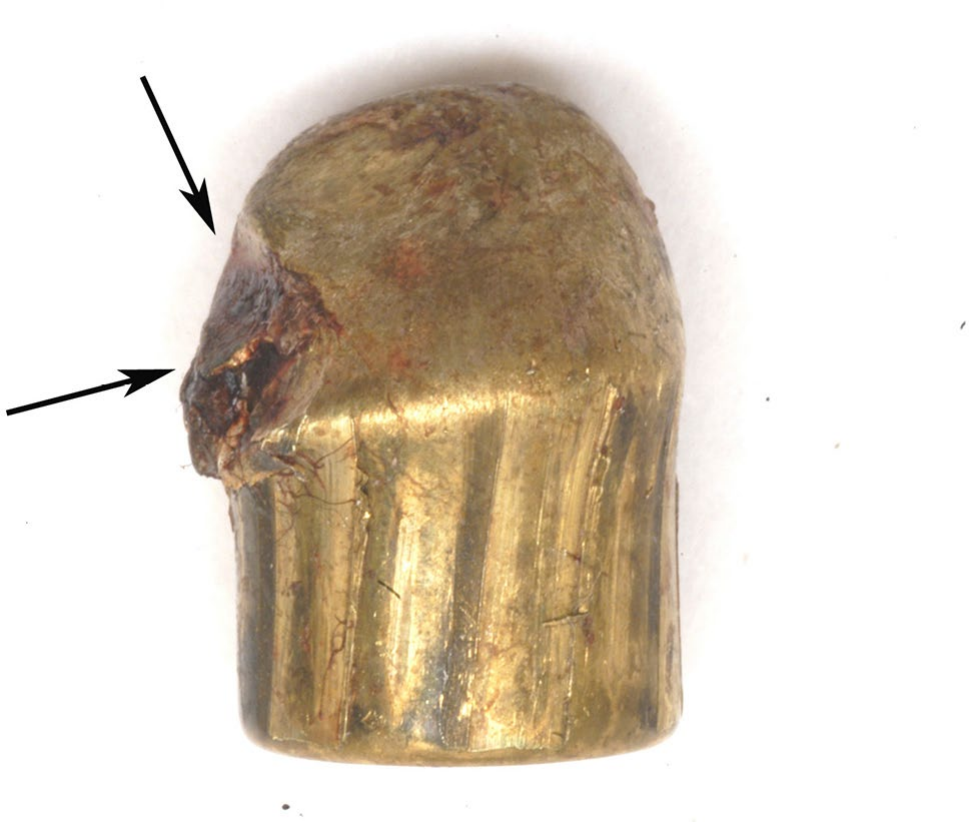
Cal. 9-mm bullet retrieved during crime scene investigation, characterized by a deformation produced by the contact with the metal wire fence (upper arrow) and the glass (lower arrow)

A man sitting in the back seat of a moving car was accidentally shot by a bullet fired from the opposite side of a highway. During the crime scene investigation, the metal wire fence placed between the two carriageways was examined, and a small, yet noticeable deflection was documented and sampled (Fig. 1a). There was a hole on the left rear window of the car with radial cracks in the glass (Fig. 1b). The victim was found in a sitting position with two GSWs to the neck, one on his left side and one on the right side, still wearing a necklace chain with two breaks at the level of the GSWs (Fig. 2a). The bullet, once retrieved during site examination, was found to be partially deformed at the ogive with a damaged jacket (Fig. 3). The event’s dynamics was initially reconstructed from the testimonies collected by bystanders who saw a man shooting from the opposite side of the highway, where a cartridge case was found.

Based on the conducted investigations, it was postulated that the bullet had impacted the metal fence with subsequent deviation from its original trajectory (first intermediate target), the rear window with the fragmentation of the glass (second intermediate target), and, lastly, the necklace worn by the victim (third intermediate target) before reaching his neck and producing a perforating GSW to the neck. For the sake of this analysis, before providing a description of the injuries caused by the deviated and deformed bullet, each of the intermediate targets will be examined.

#### First intermediate target—metal wire fence

During the crime scene investigation, the judicial police recognized that the wire fence dividing the two lanes had acted as an obstacle to the projectile's motion. A deformation was detected on the central portion of one of the cylindrical elements, orthogonally everted with respect to the plane of the wire and curved towards the side where the car was located (Fig. 1a).

According to the reconstructions, the bullet drew this portion of the intermediate target causing a glove-like semi-invagination, and then deviated to the left of the wire as suggested by the morphology of the impression, which was compatible with the cylindrical-ogival shape of the deforming agent and limited to the contact area (Fig. 3).

#### Second intermediate target—glass of the rear window

By analyzing the shape of the hole in the glass of the rear window of the car, it was postulated that an impact with the lateral surface of the bullet had occurred. The morphology was attributed both to the perturbation of the projectile’s trajectory after contact with the wire fence, and to the convexity of the glass itself (Fig. 1b).

#### Third intermediate target—necklace

Two breaks with deformed margins were found on the necklace: the one on the left side of the neck had produced some metallic fragments which deposited on the subcutaneous tissue of the cervical region, while the one on the right had branching-out metallic fibers, hence confirming the correct positioning with respect to the entrance and exit wounds (Fig. 2b–c). The bullet had caused an initial bending of the necklace, followed by tearing of the folded area, defeating the elastic capacity of the necklace.

Field emission gun–scanning electron microscopy (FEG-SEM) and scanning electron microscopy–energy dispersive spectroscopy (SEM–EDS) analysis were conducted on the necklace’s fragments and allowed the investigators to document additional blood traces at the level of the right break.

#### Final target—the victim

**Fig. 4:**
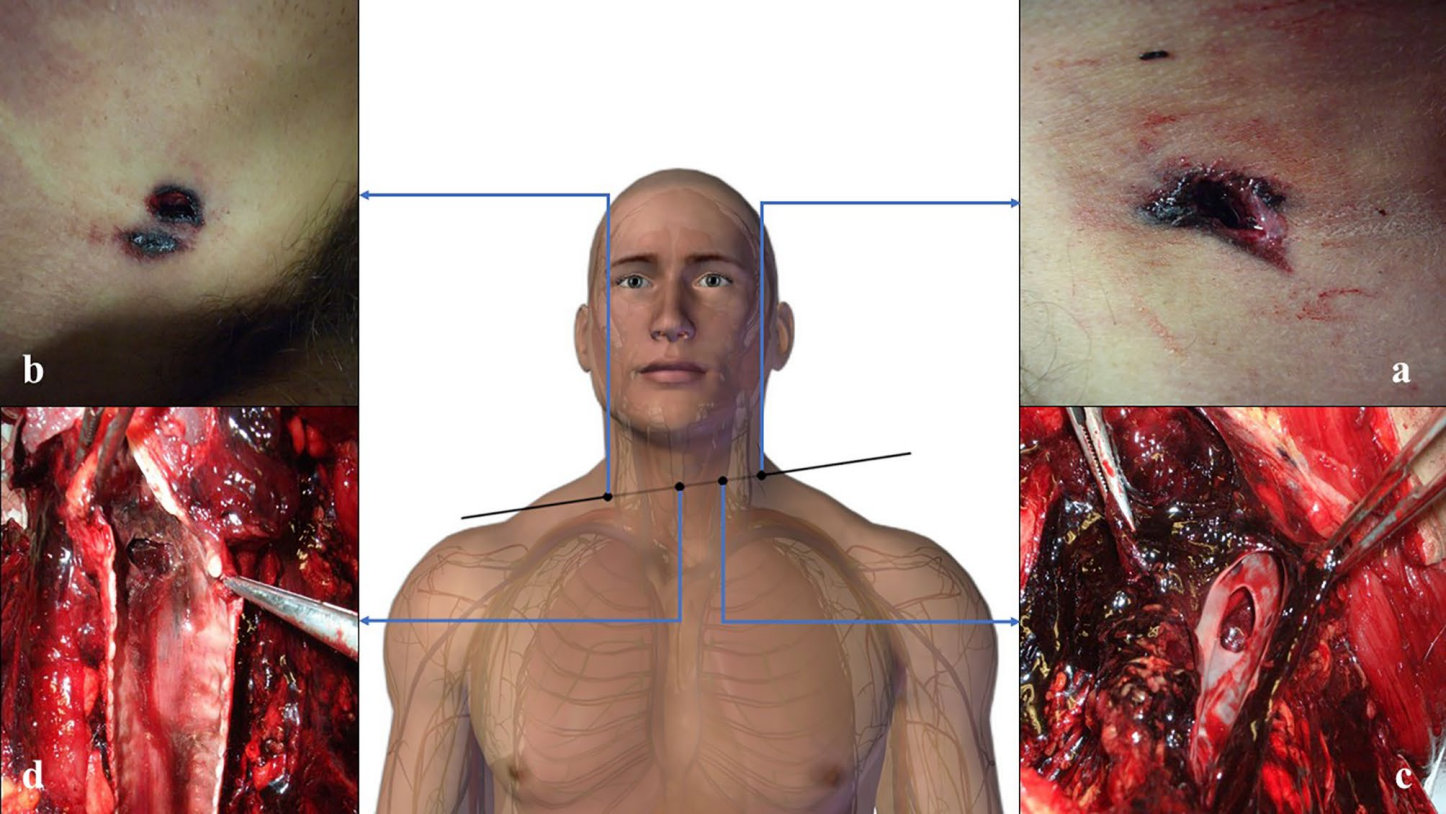
Case 1 GSWs. **a** D-shaped entrance wound produced by the lateral projection of the bullet coupled to the cutaneous fold of the neck region. **b** Jugular vein with a D-shaped hole. **c** D-shaped hole in the trachea, which caused bleeding and bronchoaspiration of blood. **d** Shored exit wound produced by the presence of the necklace. All the pictures have been oriented as follows: TOP–BOTTOM, LEFT–RIGHT. Note that some of the structures have been partially displaced during the autoptic examination to exhibit and document the wounds

A D-shaped 1.3 × 0.4 cm entrance wound was found in the left latero-cervical region of the neck, and was surrounded by a slightly depressed, triangularly shaped abrasion collar and a brown-to-black oval contusion ring (Fig. 4a). On the internal side of the cutaneous layers, golden metallic fibers were visible.

On the right side of the neck, 1 cm above the clavicle, a 1 × 0.4 cm oval exit wound was detected, which was surrounded by a small abrasion collar-contusion ring complex (Fig. 4b).

**Fig. 5:**
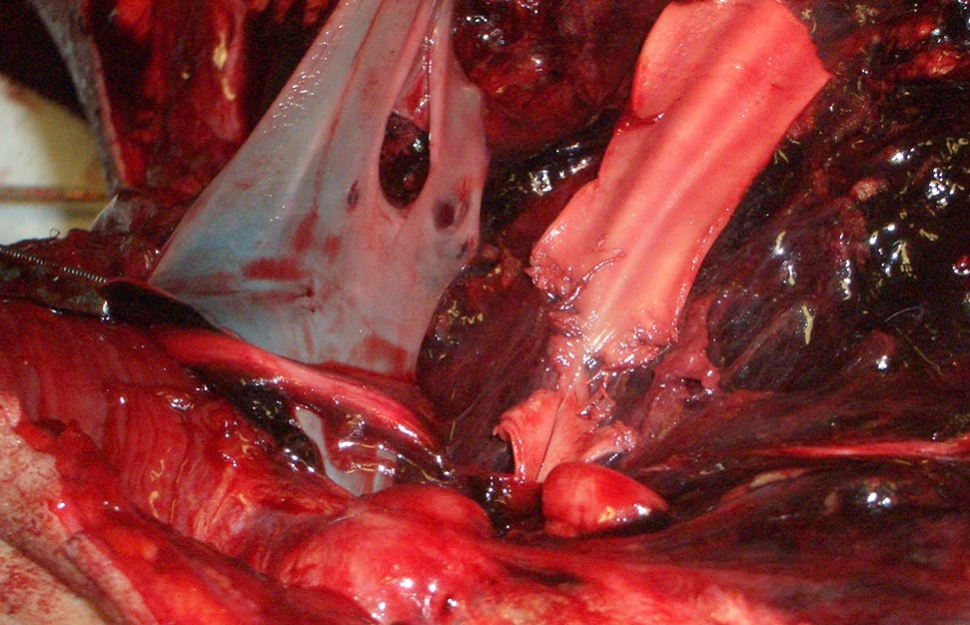
Lacerated carotid wall compared to the venous wall

Anterior and lateral neck dissection revealed a D-shaped hole in the middle third of the left jugular vein, perfectly reproducing the bullet’s lateral surface, where the tip was cranially oriented (Fig. 4c). The ventral wall of the left common carotid artery was lacerated with irregular margins (Fig. 5). Another D-shaped hole was detected at the level of the trachea, just beneath the cricoid cartilage (Fig. 4d). Both the lungs were pink with multiple red-to-purple polygonal punctuations on the pleural surface, which were compatible with bronchoaspiration.

Based on the autoptic findings, it was possible to accurately reconstruct the intrasomatic bullet course: left cervical skin, left infrahyoid muscles, left jugular vein, left common carotid artery, trachea, right intrahyoid muscles, right cervical skin.

#### Bullet’s characteristics

The 7.40 g, cal. 9 Parabellum bullet was analyzed (Fig. 3):

- The tip of the bullet was deformed and flattened by the impact with the glass of the rear window;
- The latero-ogival region of the base of the bullet was curved, as a result of the impact with the wire fence, which was also demonstrated by SEM–EDS analysis;
- The analysis on the bullet’s surface also detected the presence of silver and cadmium residues, which were the main components of the necklace;
- Additionally, silica fragments from the rear window were present on the projectile’s ogive; therefore, contact with the intermediate targets was confirmed.

### Case 2

#### The incident details and crime scene investigation

A family of three was assaulted by two robbers while walking on a sidewalk. A single bullet was fired from a revolver and struck both the man and his 11-month-old daughter. The emergency response system was activated, and cardiopulmonary resuscitation was performed, without any successful restoration of consciousness nor perfusion of the victims.

When the medical examiner arrived, the man was leaning on his right side on the sidewalk, while the infant had been moved into the ambulance by the rescuers for the resuscitation maneuvers. Several blood traces were found and documented during the site inspection. The right half of the infant’s face was covered in blood, with diffuse blood traces on her clothes. A D-shaped GSW was detected at the level of the sternum of the man’s chest, with no evidence of an exit wound. No cartridge case was retrieved during the crime scene investigation.

#### Intermediate target—the infant’s head

**Fig. 6:**
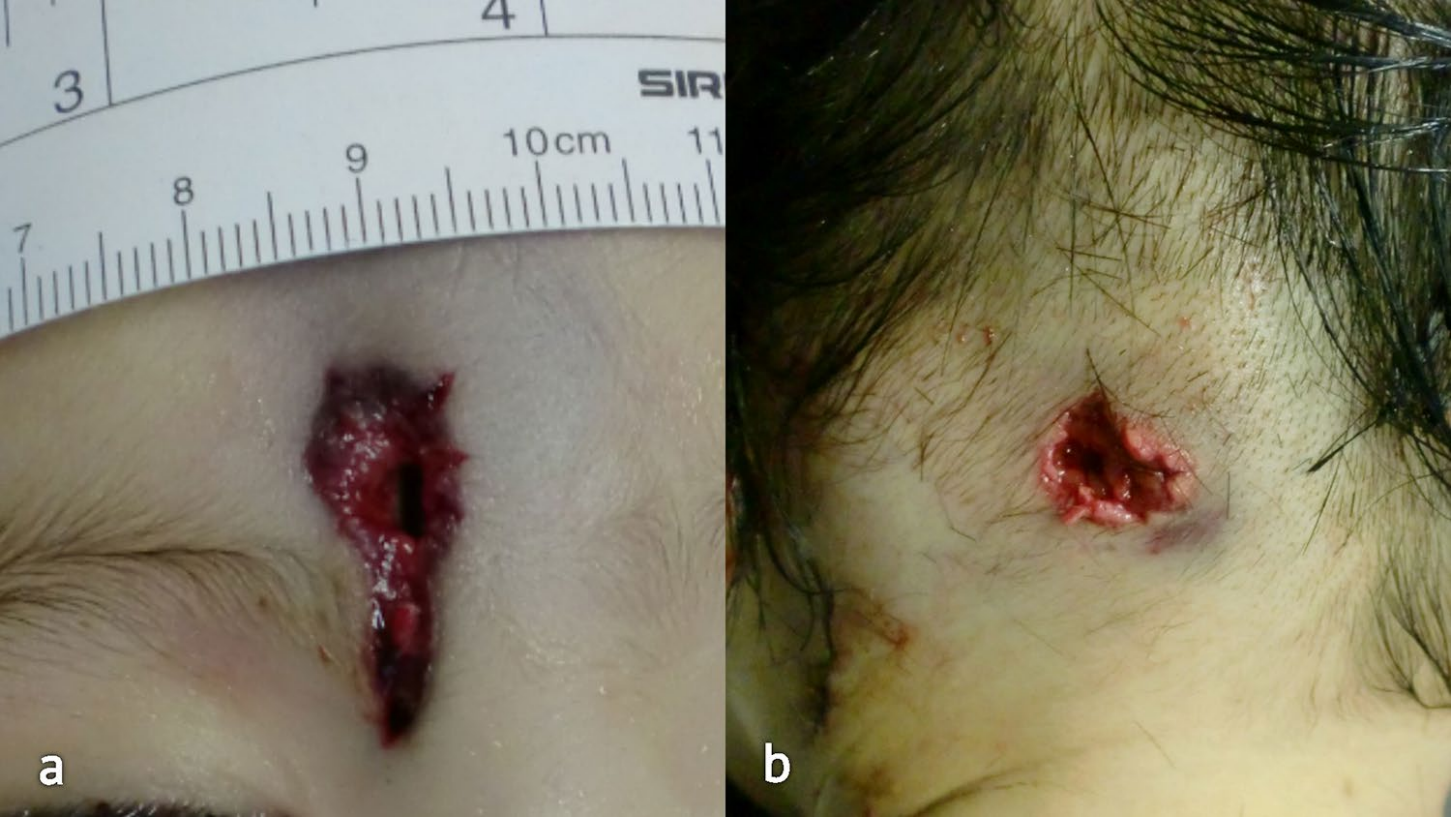
Case 2 intermediate target. **a** Entrance wound on the forehead of the infant, with a prominent V-shaped projection. **b** Irregular exit wound on the occipital region

Upon removal of the blood from the infant’s forehead, a 0.6 cm diameter round entrance wound with a prominent V-shaped projection surrounded by an abrasion collar with a contusion ring was revealed on the superior portion of the glabella (Fig. 6a). On the left occipital region, a round exit wound, 0.5 cm in diameter, was detected (Fig. 6b).

In the occipital region, the internal surface of the scalp was diffusely infiltrated by blood, while the frontal bone had a 0.8-diameter hole. The two wounds were connected by an intrasomatic bullet path, with a ventro-dorsal, right-to-left, top-to-bottom bullet course. The left frontal lobe, the left temporal lobe, and the left cerebellar lobe were collapsed with diffuse parenchymal and subarachnoid hemorrhage accompanied by cerebral edema. The skull was affected by a comminuted cranial base fracture with fragment dislocation and fracture lines extending up to the temporal and occipital bones.

#### Final target—the victim

A chest and abdomen X-ray was conducted prior to the autopsy and highlighted the presence of a semiconical radiopaque object with an ogival tip localized on the right side of the twelfth thoracic vertebra.

**Fig. 7:**
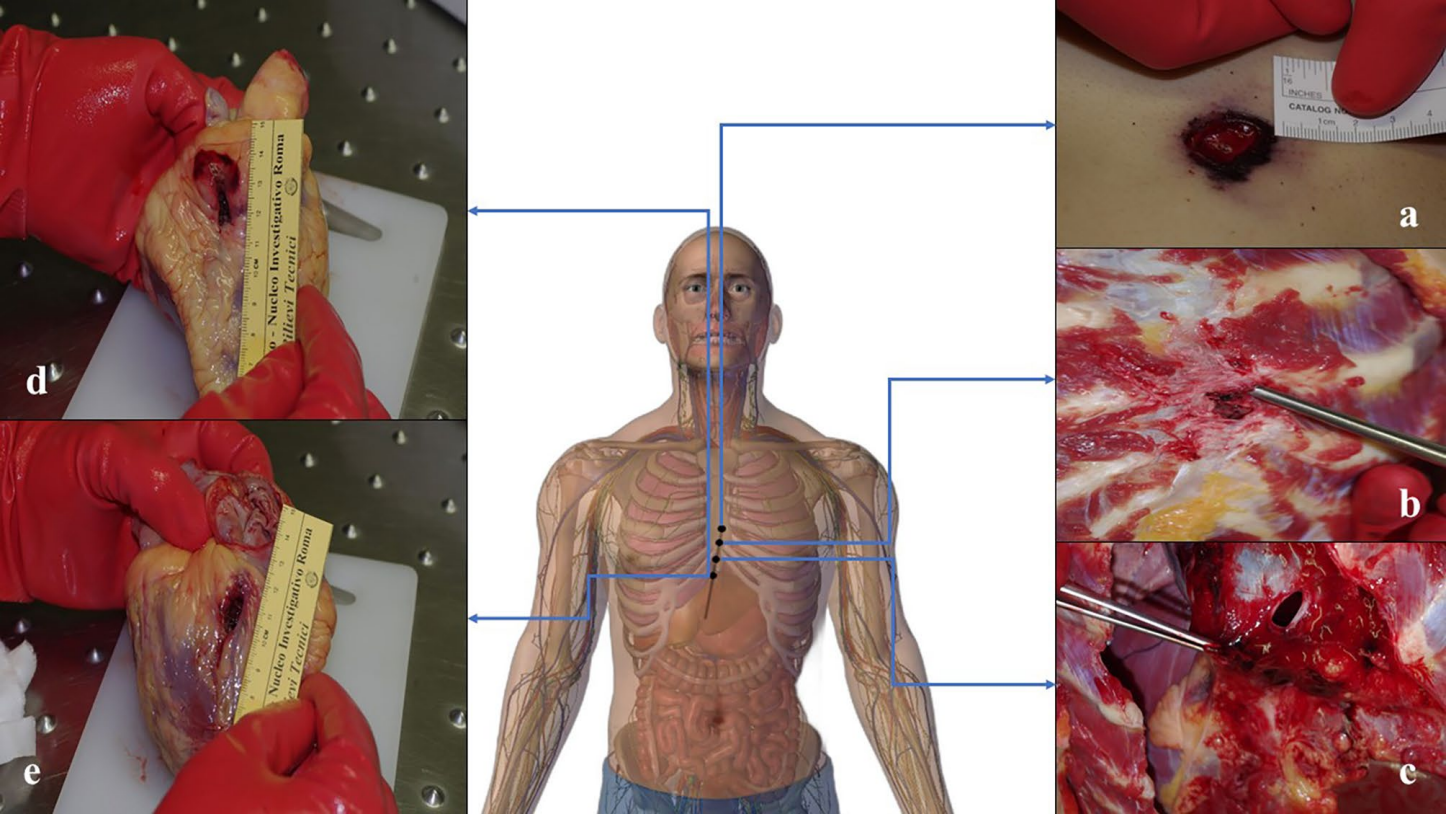
Case 2 GSWs. **a** D-shaped entrance wound on the sternal surface of the thorax. **b** Removed sternum with an irregularly shaped hole and bony fragments. **c** D-shaped hole on the pericardial sac. Anterior **d** and posterior **e** cardiac walls with D-shaped lesions, which appear longer and thinner than the pericardial ones. All the pictures have been oriented as follows: TOP–BOTTOM, LEFT–RIGHT. Note that some of the structures have been partially displaced during the autoptic examination to exhibit and document the wounds

On the left side of the midsternal line, 10.2 cm below the left jugular notch, an oval entrance wound was detected. This wound was 1.7 × 1.2 cm in dimension and was surrounded by an abrasion collar with a contusion ring (Fig. 7a).

A 1 × 0.5 cm irregularly shaped wound was documented on the body of the sternum at the level of the IV intercostal space. Fragments from the outer layer of the sternal bone were detected together with three black metallic fragments embedded in the trabecular bony tissue.

Upon removal of the sternum, osseous fragments protruding towards the internal thoracic cavity were detected around a 1.2 × 0.6 cm wound (Fig. 7b). In the intraosseous bullet path, an additional black metallic fragment was retrieved. On the parietal surface of the pericardium, a D-shaped GSW was documented and massive hemopericardium was present (Fig. 7c).

Two similar wounds were detected both on the anterior and the posterior walls of the right ventricle (Fig. 7d–e).

On the diaphragmatic surface of the pericardium, an irregular wound was documented, with signs of the left hepatic lobe involvement. A small round lesion was detected on the lesser omentum and a cal. 9-mm bullet was found in the peritoneal cavity, at the level of the posterior surface of the right hepatic lobe.

Therefore, the intrasomatic bullet path was reconstructed: left midsternal line, body of the sternum, pericardial sac, anterior surface of the right ventricle, posterior surface of the right ventricle, diaphragmatic surface of the pericardial sac, left hemidiaphragm, left hepatic lobe, lesser omentum, epiploic retrocavity (XII thoracic vertebral body). The bullet course had an antero-posterior, left-to-right, up-to-down direction.

#### Bullet’s characteristics

The cal. 9-mm bullet retrieved during the autopsy showed no signs of deformation nor fragmentation, with an intact jacket.

## Discussion

The interaction between the bullet and an intermediate target can result in peculiar GSWs which reflect the projectile’s behavior in space. In the two cases presented in this article, the entrance wounds’ morphology was characterized by a D-shape appearance, which was first described in 1984 by Donoghue et al. [8] and is produced by the impact of the lateral surface of the bullet with the cutaneous surface [2].

In case 1, the projectile’s course was first altered by the metallic wire fence, producing the well-known phenomenon of ricochet which occurs when the angle of incidence between the two objects is small, resulting in a tangential deviation from the original course [7]. In this case, the contact with this intermediate target led to the dispersion of kinetic energy which was later accentuated by the impact with the rear window and the necklace worn by the victim. Specifically, the metal chain also produced an asymmetric abrasion collar and contusion ring, which was more prominent along the minor axis of the entrance wound. Moreover, the anatomical district itself, i.e., the cutaneous fold of the neck, contributed to the final appearance of both the entrance hole and the surrounding skin. For the exact same reasons, the exit wound showed similar findings when compared to the entrance, as while exiting the skin the bullet encountered the other portion of the necklace, producing a shored exit wound [12].

In case 2, the D-shaped conformation was observed in the final target, i.e., the father, where the destabilizing factor was another human being that was trespassed and acted as an intermediate target: the bullet perforated the infant’s head and lost most of its kinetic energy, but still retained the capacity to cause damage and penetrate in the man’s chest. In this case, an additional element of interest is the comparison between the two entrance wounds which are both atypical but are the result of two different physical processes: the lesion on the infant’s head was produced by a stable bullet and had a long V-shaped projection because of the properties of the skull and the overlying scalp, while the lesion on the man’s chest is represented by a wobbling, destabilized bullet that had changed its orientation in space [3].

In forensic literature, the D-shaped morphology has been described at the level of the cutaneous surfaces, while in the present case reports some structures throughout the intrasomatic bullet path were also characterized by the same finding. The study of the internal wounds warrants a few considerations for what concerns the physical characteristics of the anatomical districts.

In both the incidents presented in this article, the destabilized bullet produced the same results in terms of wounding effects, where the distribution and the morphology of the GSWs varied according to the different anatomical compartments.

First, structures like muscles and arterial wall in case 1, bone and liver in case 2, showed irregularly shaped wounds, leaving no traces that could reveal information about the bullet’s behavior. These tissues have limited stretch capacity in the case of GSWs and they were lacerated with fragmented margins, leading to a non-specific appearance of the wound.

On the other hand, the venous wall and the trachea in case 1, the pericardial sac in case 2, led to the production of the D-shaped morphology and allowed the examiner to clearly reconstruct the path, the orientation, and the rotation along its axis of the bullet throughout its course. These structures are more compliant to the energy and the displacement exerted by the bullet, especially when the velocity and therefore the temporary cavity effect are low. The GSWs at the level of the right ventricle were somewhat similar, where the D-shaped morphology could still be recognized despite the tissue recoil and the greater thickness when compared to the pericardium, which led to a less definite appearance of the bullet’s lateral surface.

The different behavior of the different anatomical districts varied according to the intrinsic mechanical properties of the structures and the thickness of the tissues drawn by the bullet. The jugular vein, the trachea, and the pericardium have thin elastic walls, which make them more compliant to the energy and the displacement exerted by the bullet, especially when the velocity and therefore the temporary cavity effect are reduced or neglegible. Instead, other vascular structures, like the carotid artery are thick, semi-rigid tubes.

Notably, the D-shaped morphology was seen exceptionally well at the level of the pericardium, perfectly reproducing the bullet’s impression on the thin fibrous tissue composing this membrane.

The discovery of the D-shaped morphology both upon external inspection and autoptic examination confirmed and supported the dynamics of wound production, which had been studied extensively by the crime scene investigation and FEG/EDS-SEM analyses and allowed the forensic pathologist to postulate that in both cases interaction with intermediate targets had occurred producing significant bullet destabilization with change in orientation and velocity.

## Conclusion

Regardless of the circumstantial evidence, a D-shaped morphology can accurately suggest that the bullet’s behavior has been altered by a significant impact with one or more intermediate targets before reaching the final target. Isolation and sectioning of the anatomical structures coupled to the study of the lesion(s) during the autopsy can lead to the direct visualization of D-shaped GSWs throughout the bullet’s path and give additional information on the stability of the projectile. There is no unique mechanism of destabilization, since the interaction with interposed objects can result in different phenomena; therefore, the correlation between crime scene investigation and autoptic findings is crucial in the determination of the event’s dynamics.

## Key points

1. The interaction between a bullet and one or more intermediate target(s) can produce atypical entrance wounds.
2. The D-shaped morphology has been described exclusively on the skin in entrance wounds
3. In the two cases presented in this article, D-shaped wounds were observed throughout most of the intrasomatic bullet path as well as on the skin.
4. The presence of intrasomatic D-shaped wounds during the forensic examination can suggest that interaction with an intermediate target has occurred.

## Data Availability

N/A.

## Materials and/or code availability

N/A
